# Development and Comparative Evaluation of Two Enzyme-Based Amperometric Biosensor Designs for Alanine Aminotransferase Determination in Biological Fluids

**DOI:** 10.3390/mi16101168

**Published:** 2025-10-15

**Authors:** Daryna Mruga, Yevhen Vakhovskyi, Veronika Bakhmat, Viktoriya Pyeshkova, Sergii Dzyadevych, Oleksandr Soldatkin

**Affiliations:** 1 Institute of Molecular Biology and Genetics, National Academy of Sciences of Ukraine, 150 Zabolotnoho Str., 03680 Kyiv, Ukraine; 2 ESC “Institute of Biology and Medicine”, Taras Shevchenko National University of Kyiv, 64 Volodymyrska Str., 01003 Kyiv, Ukraine; 3 School of Engineering, University of Warwick, Coventry CV4 7AL, UK; 4 Educational Scientific Institute of High Technologies, Taras Shevchenko National University of Kyiv, 64 Volodymyrska Str., 01003 Kyiv, Ukraine; 5 Faculty of Biomedical Engineering, National Technical University of Ukraine “Igor Sikorsky Kyiv Polytechnic Institute”, 37 Beresteysky Avenue, 03056 Kyiv, Ukraine

**Keywords:** alanine aminotransferase, pyruvate oxidase, glutamate oxidase, amperometry, biosensor, enzyme, ALT activity

## Abstract

Alanine aminotransferase (ALT) is a key biomarker of liver function. Compared with conventional assays for ALT detection—which are expensive, time-consuming, labor-intensive, and require experienced personnel—biosensors represent a promising alternative, but it remains unclear which biorecognitive enzymatic configuration offers the best analytical performance for ALT detection. This study presents the development and comparative evaluation of two amperometric biosensors based on oxidase biorecognition elements: pyruvate oxidase (POx) and glutamate oxidase (GlOx). Enzymes were immobilized onto platinum electrodes under optimized conditions using entrapment for POx (pH 7.4, enzyme loading 1.62 U/µL, PVA-SbQ concentration 13.2%) and covalent crosslinking for GlOx (pH 6.5, enzyme loading 2.67%, glutaraldehyde concentration 0.3%). Analytical parameters were systematically assessed, including linear range (1–500 U/L for POx vs. 5–500 U/L for GlOx), limit of detection (1 U/L for both), and sensitivity (0.75 vs. 0.49 nA/min at 100 U/L). The POx-based biosensor demonstrated higher sensitivity and lower detection limits, whereas the GlOx-based biosensor exhibited greater stability in complex solutions and reduced assay costs due to a simpler working solution. Moreover, while the POx-based system is uniquely suited for ALT determination, the GlOx-based sensor can be affected by AST activity in samples but may also be adapted for targeted AST detection. Overall, the study highlights a trade-off between sensitivity, robustness, and versatility in ALT biosensor design, providing guidance for the rational development of clinically relevant devices.

## 1. Introduction

**Figure 1:**
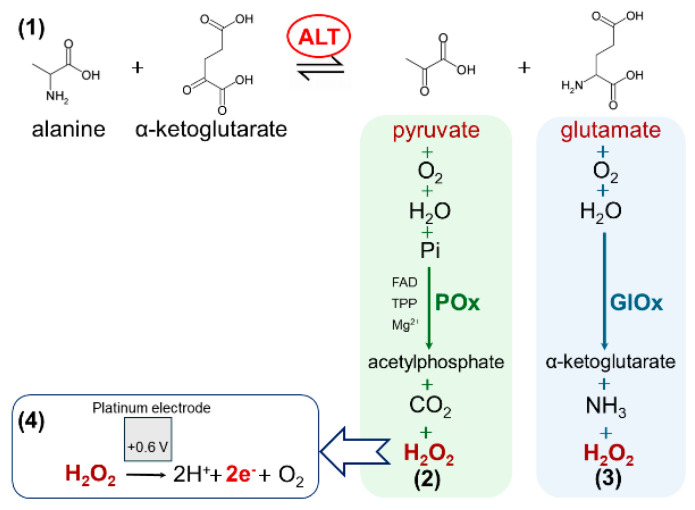
Schematic illustration of the enzymatic reactions and detection principle of ALT activity using POx- and GlOx-based amperometric biosensors.

Alanine aminotransferase (ALT, EC 2.6.1.2) is an intracellular enzyme that catalyzes the reversible transamination between L-alanine and α-ketoglutarate, producing pyruvate and L-glutamate (Figure 1, reaction 1) [1]. This reaction is part of several key metabolic cycles in the human body, including the tricarboxylic acid cycle, the glucose–alanine cycle, amino acid metabolism, and nitrogen balance through the urea cycle [2,3]. Due to its localization, ALT serves as a highly sensitive and relatively specific biomarker for liver health. In healthy people, ALT levels are usually below 30 U/L. When liver cells are damaged due to diseases like hepatitis, liver cirrhosis, or fatty liver disease, ALT is released into the blood, and its level can increase significantly, sometimes by 8 to 35 times more than normal [4,5,6].

To date, several methods for the quantitative determination of ALT activity have been developed, including colorimetric, spectrophotometric, fluorescent, chemiluminescent, and chromatographic techniques [7]. These methods are highly sensitive and selective, but they often require expensive equipment, trained specialists, and complex preparation of samples. This makes them less practical for fast or portable testing, especially outside laboratories.

Therefore, there is a growing need for analytical approaches that are simpler, faster, more cost-effective, and suitable for point-of-care applications. In this context, biosensor technologies, particularly amperometric biosensors, offer significant advantages, including lower cost, portability, and the potential for point-of-care testing. These features make them a promising alternative to traditional laboratory-based techniques for the determination of the activity of different enzymes, including ALT [8,9,10,11,12].

ALT itself cannot be detected directly by electrochemical biosensors, because it has no strong electroactive properties. Therefore, ALT activity (amount of enzyme required to convert 1 μmol of substrate to product per minute) is measured through its reaction products, such as glutamate or pyruvate, that are detected using special enzymes. One of these special enzymes is pyruvate oxidase (POx), used in works [13,14], which reacts (Figure 1, reaction 2) with pyruvate to form hydrogen peroxide, a compound that can be measured using amperometry. Another more frequently used option is glutamate oxidase (GlOx), as reported in [15,16], which reacts (Figure 1, reaction 3) with glutamate producing hydrogen peroxide. When hydrogen peroxide reaches the platinum electrode, it breaks down (Figure 1, reaction 4) at an applied potential (about +0.6 V), generating electrons and changing the electric current. A recent review summarizes the prevalence, typical architectures, and main limitations of GlOx- and POx-based ALT biosensors, highlighting current trends toward improved stability and multiplexing in clinical applications [17].

Although both GlOx- and POx-based biosensors have been employed for ALT activity measurement, a direct comparative analysis between them has not yet been reported. Clarifying which enzyme provides better sensitivity, stability, and practical usability as a bioselective element is essential for advancing ALT biosensor development for clinical diagnostics.

In this study, we constructed two types of amperometric biosensors for ALT detection using GlOx and POx. Their fabrication procedures (which should be as simple as possible, involving the fewest steps and minimal use of toxic reagents to facilitate potential scale-up), operating parameters (where complex multicomponent solutions or extreme conditions are impractical for routine use), and analytical performance (critical for assessing accuracy and the applicable measurement ranges) were systematically evaluated. Our goal was to evaluate both bioselective systems under highly comparable conditions, using identical instrumentation, electrodes, buffers, and sensor modifications, in order to reveal the fundamental characteristics, advantages, and limitations of each enzymatic system, thereby allowing the determination of which biosensor design is more effective and better suited for further optimization and clinical application.

## 2. Materials and Methods

### 2.1. Materials

Pyruvate oxidase (POx, 35 U/mg) from *Aerococcus viridans* and alanine aminotransferase (ALT, 84 U/mg) from porcine heart were purchased from Sigma-Aldrich (St. Louis, MO, USA). Recombinant glutamate oxidase (GlOx, 7 U/mg) from *Streptomyces* sp. was obtained from Yamasa Corporation (Araoicho, Japan). HEPES, glutaraldehyde (GA), meta-phenylenediamine, sodium pyruvate, sodium glutamate, magnesium nitrate, thiamine pyrophosphate (TPP), pyridoxal phosphate (PLP), α-ketoglutarate, and alanine were from Sigma-Aldrich. Interferent reagents, such as ascorbic acid, dopamine hydrochloride, citric acid, urea, methionine, arginine, lysine, cysteine, asparagine, glucose, acetaminophen, NaCl, KCl, NaN_3_, and CaCl_2_, were also purchased from Sigma-Aldrich. Polyvinyl alcohol with steryl pyridinium groups (PVA-SbQ) was obtained from Toyo Gosei Kogyo Co. Ltd. (Chiba, Japan). All other inorganic reagents, including hydrogen peroxide, ethanol, and potassium dihydrogen phosphate, were of domestic reagent-grade quality.

### 2.2. Amperometric Equipment

Amperometric measurements were performed using a standard three-electrode system consisting of a PalmSens potentiostat (Palm Instruments BV, Houten, The Netherlands), an 8-channel multiplexer, and several platinum disc working electrodes, a platinum counter electrode, and an Ag/AgCl reference electrode [18].

### 2.3. PPD Membrane

Amperometric sensors are sensitive to numerous electroactive compounds; therefore, minimizing interference from molecules such as ascorbic acid is essential for accurate serum measurements. This was addressed by modifying the platinum electrode with a semi-permeable poly (meta-phenylenediamine) membrane, previously reported as effective for improving selectivity toward hydrogen peroxide [19]. The membrane’s pore size permits H_2_O_2_ diffusion while blocking larger molecules. Electrochemical polymerization was performed on polished, ethanol-cleaned electrodes immersed in 5 mM meta-phenylenediamine in 10 mM phosphate buffer (pH 6.5) under cyclic voltammetry (0–0.9 V, step 0.005 V, rate 0.02 V/s, starting potential 0 V). Completing surface coverage was assumed after 10–20 cycles by stable voltammograms and confirmed using SEM (see Supplementary Figures S1 and S2).

### 2.4. Methods of Manufacturing Bioselective Membranes

For POx immobilization, an enzyme gel containing 10% glycerol, 5% BSA, and 4.86 U/µL POx in 25 mM HEPES buffer (pH 7.4) was prepared to enhance membrane elasticity and reduce enzyme leaching. The gel was mixed 1:2 with 19.8% PVA-SbQ photopolymer, resulting in the following mixture parameters: 3.3% glycerol, 1.67% BSA, 1.62 U/µL POx, 13.2% PVA-SbQ. Next, 0.15 μL of the mixture per electrode was applied to the electrode surface and photopolymerized under UV light (365 nm) until 2.4 J (~8 min). Electrodes were rinsed 2–3 times for 3 min in the working buffer before measurements.

GlOx was immobilized via covalent crosslinking with glutaraldehyde (GA). A gel was prepared in 100 mM phosphate buffer (pH 6.5) containing 10% glycerol, 4% BSA, and 8% GlOx. It was mixed with a 0.5% GA solution in a 1:2 ratio, resulting in the following mixture parameters: 3.3% glycerol, 1.3% BSA, 2.67% GlOx, 0.3% GA. Next, 0.05 μL of the mixture per electrode was deposited on the electrode surface, followed by air-drying for 35 min. Electrodes were also rinsed.

**Figure 2:**
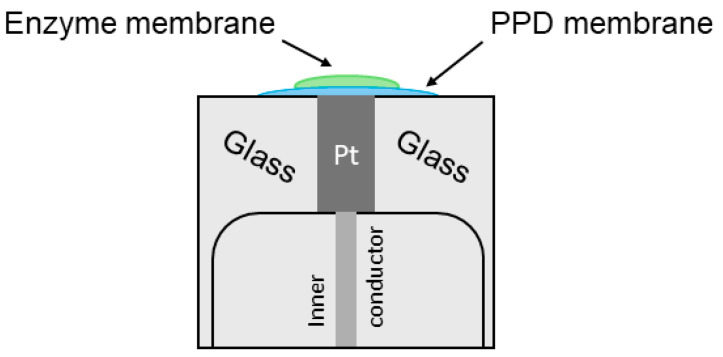
Schematic illustration of the electrode modifications.

After manufacturing, the general sensor modification appears as shown in Figure 2 and the SEM photo is shown in Figure S3.

After preraparation and in between measurements biosensors were stored in a dry state in a refrigerator at 8 °C.

Other immobilization methods tested included covalent crosslinking with glutaraldehyde (GA) at room temperature (2–40 min) or in a refrigerator (8 °C, 1–4 h). For this procedure, the enzyme gels were applied to the working electrode surface in a volume of 0.05 μL per electrode. Subsequently, the electrodes were placed in a 2 L glass desiccator containing 200 mL of 25% GA, providing an effective surface area of approximately 95 cm^2^. The final step of immobilization involved rinsing the sensors with working buffer to remove unbound molecules.

### 2.5. Methodology for Biosensor Measurement of ALT

Measurements were conducted in a 2 mL stirred cell at room temperature. A potential of +0.6 V vs. Ag/AgCl was applied to the working electrodes, which is a well-established optimal potential for H_2_O_2_ oxidation on platinum and was additionally confirmed in our experiments (see Supplementary Figure S4). Aliquots of reagents were added sequentially in a defined order, and measurements were taken after baseline stabilization. Data analysis was performed by selecting a linear portion of the biosensor response, subtracting baseline noise, and calculating the current change over 60 s as ΔI/Δt, where ΔI is the current difference and Δt is the measurement time interval.

### 2.6. Statistical Treatment

All experiments were performed at least in triplicate, with each experiment comprising three working electrodes (*n* = 9). Data analysis was carried out using OriginPro 8.5, which applies standard mathematical procedures, including linear regression, arithmetic mean, sample standard deviation, and the Hill equation. For data fitting, a modified Bezier curve was used.

## 3. Results

### 3.1. Principle of Detection

For successful amperometric detection of ALT activity, it is necessary to monitor the change in the concentration of ALT reaction products (Figure 1, reaction 1), i.e., pyruvate or glutamate, in the working solution over a defined time interval. Both biorecognition elements used in this study for indirect ALT activity analysis are oxidase-type enzymes (POx and GlOx); accordingly, each catalyzes the oxidation of one of the ALT products with the formation of hydrogen peroxide (Figure 1, reactions 2,3). Considering the cascade of reactions underlying the biosensors, the rate of hydrogen peroxide generation is directly proportional to the rate of ALT product formation and thus reflects ALT activity. Hydrogen peroxide is subsequently oxidized at the surface of the amperometric transducer (Figure 1, reaction 4), which is recorded as an increase in current. This current rise occurs continuously and linearly with the increasing concentration of the intermediate analyte (pyruvate for POx or glutamate for GlOx) generated by ALT activity. Therefore, the biosensor response is calculated as the change in current per unit time.

It was confirmed that the observed current change originates from the enzymatic reaction cascade, as evidenced by the following:

- Electrodes without enzyme-containing membranes showed no response to the presence of all reagents (including ALT) in the working buffer, maintaining a stable baseline current.
- Biosensors in the absence of ALT also exhibited no response to the complete reagent mixture, retaining a stable baseline current.

### 3.2. Immobilization Processes

**Figure 3:**
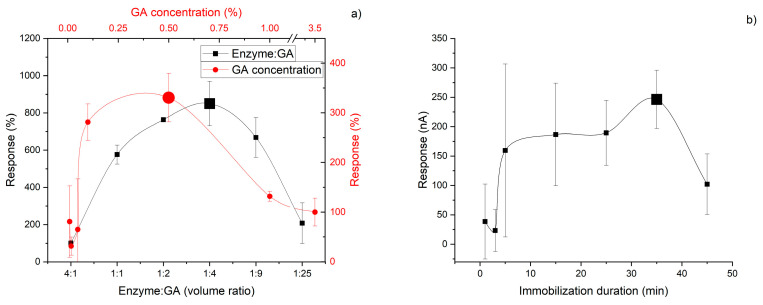
Dependence of the GlOx-based biosensor responses on immobilization parameters: glutaraldehyde (GA) concentration and the volume ratio of GA to enzyme gel (**a**), and on immobilization time (**b**).

Different immobilization methods were selected for GlOx and POx: covalent crosslinking and entrapment, respectively. During the optimization of GlOx immobilization, several approaches were tested, including entrapment in a photopolymer and crosslinking in GA vapors. However, the most successful results were obtained when the enzyme was immobilized in a GA droplet. This compound is capable of forming covalent Schiff-base bonds between protein molecules. In the course of optimizing GlOx immobilization on the platinum electrode surface, the optimal concentration of the crosslinking agent, the ratio of crosslinker to enzyme gel solutions, and the immobilization duration in air at RT were determined (Figure 3). All parameters exhibited a bell-shaped dependence with a clear optimum under certain conditions.

**Figure 4:**
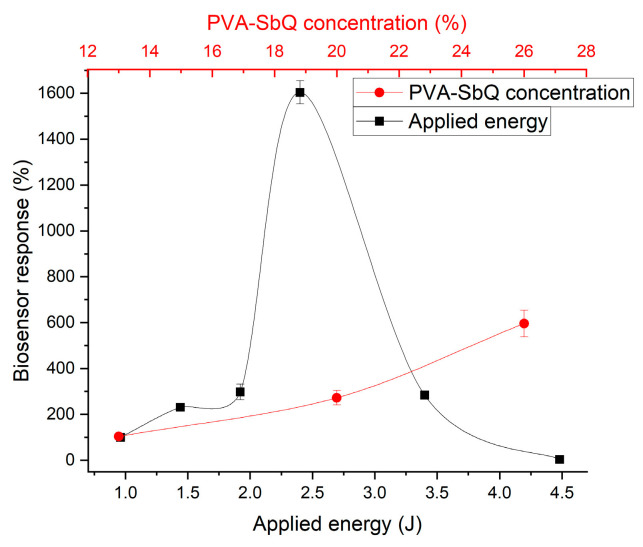
Dependence of the POx-based biosensor responses on irradiation intensity and PVA-SbQ concentration.

Originally, crosslinking was also considered for POx, as this method typically forms strong bonds and a robust membrane (immobilizations in GA droplets at RT and at 4 °C, immobilization in GA vapors at RT were tested). However, the results obtained were unsatisfactory: the enzymatic activity dropped drastically after immobilization, responses were irreproducible, and no stability was observed. Therefore, a milder method was chosen, namely entrapment in the PVA-SBQ photopolymer. Entrapment involves the in situ formation of a polymer matrix around enzyme molecules, physically restricting their movement. This method yields a somewhat less mechanically stable membrane but avoids alterations of protein conformation and deformation of the active site, which is particularly relevant for POx. During the optimization of immobilization conditions, the optimal UV-light intensity and PVA concentration were determined for POx (Figure 4). A PVA concentration of 20% was chosen as the working value, despite slightly better results at 26% (Figure 4), because higher concentrations resulted in excessive viscosity and density of the polymer, complicating the immobilization process.

Thus, GlOx demonstrated greater flexibility in the choice of immobilization strategy, tolerating aggressive covalent crosslinking, whereas POx proved to be a less stable enzyme that requires more delicate immobilization through polymer entrapment.

### 3.3. Media Requirements

**Figure 5:**
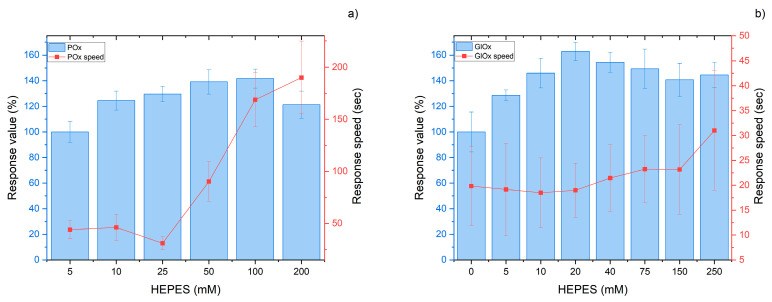
Dependence of biosensor response magnitude (bars) and response speed rate (line) for POx-based (**a**) and GlOx-based (**b**) biosensors on buffer concentration.

For the efficient performance of each enzyme (POx or GlOx) within the biorecognition element, a stable working environment is required. This is achieved using an appropriate buffer system that ensures constant pH, even when analyzing complex biological samples. Since the biosensors were designed to operate with biological fluids of pH 7–8 (e.g., blood), HEPES buffer with a matching pH range was employed. Optimization of the buffer concentration revealed that for the POx-based biosensor, the optimum concentration was 25 mM, despite the highest responses being observed at 100 mM. A concentration of 25 mM HEPES was selected because, at this value, the fastest responses were obtained while maintaining a sufficiently high response magnitude (Figure 5a). The increase in response time at higher buffer concentrations can be explained by an excessive buffering capacity, which hinders the local pH change required for catalysis at the POx active site. For the GlOx-based biosensor, the optimum HEPES concentration was 20 mM (Figure 5b). In this case, no significant increase in response time was observed by increasing the buffer capacity, unlike for POx (at 100 mM HEPES, approximately 20% for GlOx, compared to 540% for POx). This behavior may be explained in two ways: either the working pH corresponds more closely to the pH in the GlOx active site during catalysis, or GlOx carries out the catalytic process more easily and stably. The pH dependence can be supported by the fact that the optimum pH of GlOx is 7.0–7.4 [20,21], while the optimum pH of POx lies within 6.5–7.0 [22]. Thus, the optimum of GlOx is closer to the working conditions used in our experiments. Regarding catalytic efficiency, the reported kcat of GlOx is 85.8 s^−1^ [23], whereas exact values for POx from *Aerococcus viridans* are not available. However, a similar enzyme such as pyruvate:quinone oxidoreductase has a kcat of 37.8 s^−1^ [24], and based on supplier data on specific activity, the estimated kcat of POx is 35 U/mg × 66.25 kDa per subunit [25] = 38.6 s^−1^. Therefore, the catalytic activity of GlOx can be considered more than twofold higher.

**Figure 6:**
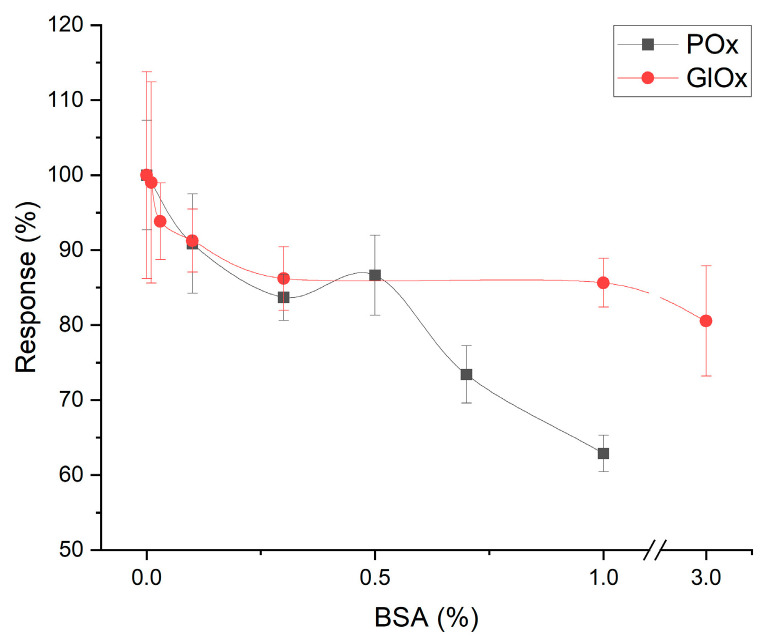
Influence of protein on the response magnitude of POx- and GlOx-based biosensors.

To evaluate the applicability of both biosensors for real biological samples, the effect of protein content of the analyzed solution on the analytical parameters was studied. The presence of large protein molecules, in particular BSA, in the working buffer negatively affected the performance of both biosensors (Figure 6). These molecules gradually contaminate the biorecognition membrane by adsorbing on its surface, thereby hindering the diffusion of substrates and reaction products and reducing response accuracy. The reference total protein concentration in human serum is approximately 120 g/L [26]. Considering the 10-fold dilution applied during analysis, the estimated impact of serum proteins on biosensor performance was estimated at 29% response decrease for the intermediate analyte (pyruvate) in the POx-based system and 10% response decrease for the intermediate analyte (glutamate) in the GlOx-based system. However, it should be emphasized that this effect develops gradually rather than immediately, and, therefore, measurement of real samples by comparing responses with a pre-obtained calibration curve remains feasible. Furthermore, rinsing of the biosensors between measurements removes a considerable amount of adsorbed proteins from the membrane. Thus, it was demonstrated that the GlOx-based biosensor is more resistant to the influence of high protein concentrations, although this factor is not critically limiting for ALT activity analysis in real samples.

**Figure 7:**
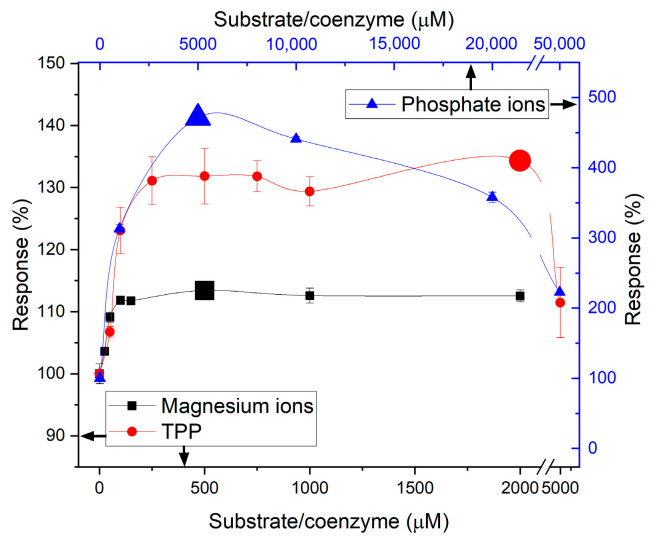
Dependence of the POx-based biosensor responses on phosphate ions, magnesium ions, and thiamine pyrophosphate (TPP) concentrations.

Another factor that can be considered a drawback of the biosensor based on POx is the need to add additional compounds to the working medium, the concentrations of which must be constantly controlled, which, to some extent, complicates the processes of development and application of this biosensor. Specifically, for the progression of the POx reaction, the presence of several substrates and cofactors is required (Figure 1, reaction 2). The affinity constants of POx substrates are not reported in the literature, but there are constants for enzymes in the same family that vary considerably and are as follows: pyruvate 0.4 mM and phosphate ions 2.3 mM [27], TPP 13 μM and magnesium ions 82 μM [28]. Accordingly, it was necessary to determine their optimal concentrations (Figure 7). Unfortunately, the optimum substrate concentrations obtained were higher than those mentioned above; however, this is a typical situation for immobilized enzymes, associated with changes in activity and diffusion characteristics. Moreover, the concentration ratios also did not match the expected values. Most likely, this can be explained by the fact that the reported optima were determined for free enzyme, whereas enzyme immobilization can significantly alter not only the required substrate concentrations but also the substrate affinity constants to the active site [29].

The selectivity of both bioselective elements towards intermediate analytes was excellent. The influence of electroactive substances was eliminated by an additional semipermeable membrane based on phenylenediamine. As a result, compounds such as cysteine and dopamine contributed up to 10% of the response to pyruvate in the POx-based biosensor. The biosensor based on GlOx showed an up to 1% response to dopamine, asparagine, citric acid, glutamine, and aspartic acid. A number of other compounds (glucose, α-ketoglutarate, acetaminophen, NaCl, KCl, NaN_3_, CaCl_2_, lysine, arginine, methionine, alanine, ascorbic acid, and urea, etc.) did not induce any response in either biosensor.

Another widely used liver biomarker, aspartate aminotransferase (AST) (an enzyme similar to ALT), did not raise any response from the POx-based biosensor either in the presence or absence of ALT. AST also did not produce any response in the GlOx-based biosensor in the absence of ALT, but in its presence, it caused a 10% increase in the response to ALT. The influence of AST on the performance of the GlOx-based biosensor, even in the absence of AST substrate, can be explained by the identical second step of the ALT [30] and AST [31] reactions, during which the amino group is transferred via the PLP coenzyme (PLP-NH_2_ complex) to a ketoacid to form glutamate. The PLP-NH_2_ complex is formed during the first step of the ALT reaction (when the amino group is detached from the amino acid), when it diffuses into the active site of AST, and immediately catalyzes the second step of the AST reaction. As a result, the rate of glutamate formation nonspecifically increases in the working cell, leading to overestimated ALT detection values in the presence of AST using the GlOx-based biosensor.

Therefore, in the course of selectivity studies of biosensor designs for ALT analysis, it was shown that both biosensors are highly selective toward the target analyte and can be applied in multicomponent fluids. However, the POx-based biosensor is more promising due to the absence of AST interference in its measurements.

### 3.4. ALT Substrates and Coenzyme Content

**Figure 8:**
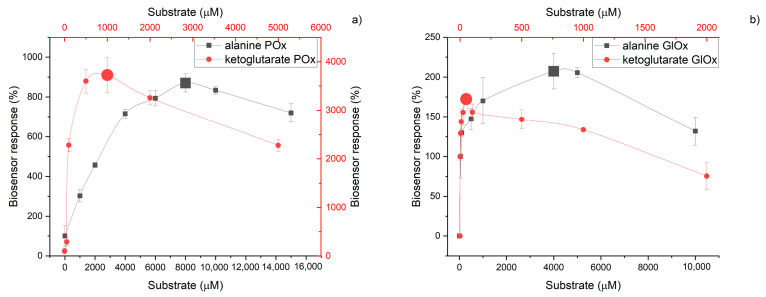
Dependence of biosensor responses on ALT substrate concentrations (alanine, α-ketoglutarate) for POx-based (**a**) and GlOx-based (**b**) systems.

Since ALT is a multisubstrate enzyme with a single active site, the optimal substrate concentrations were selected taking into account their different affinity constants for ALT (28 mM alanine and 0.4 mM ketoglutarate [32]). The study showed that the optimal substrate concentrations for ALT were higher when working with POx compared to when working with GlOx. Moreover, the ratio of alanine to ketoglutarate differed significantly between the POx- and GlOx-based biosensors, amounting to 4:1 vs. 80:1, respectively (Figure 8). Several explanations for this effect can be proposed. In particular, the ketoglutarate concentration in solution is replenished due to GlOx activity (Figure 1, reaction 3), and thus it is not depleted but maintained at a stable level. In addition, such differences in optimal conditions can be explained by the fact that proper functioning of POx requires additional reagents, namely magnesium and thiamine pyrophosphate, which are absent in the GlOx-based biosensor. Magnesium ions are known to form complexes with organic compounds (as has been shown for many central metabolites such as glutamate, aspartate, and formic acid [33]), thereby reducing substrate (particularly alanine) availability and affinity to the ALT active site. The optimal concentration of the ALT coenzyme pyridoxal phosphate (PLP) was 10 µM for POx and 50 µM for GlOx, indicating a slightly higher PLP requirement for the GlOx-based biosensor.

Thus, it can be concluded that the GlOx-based system is somewhat more cost-effective, owing to the lower substrate concentrations required in the working solution.

### 3.5. Comparison of Analytical Characteristics

**Figure 9:**
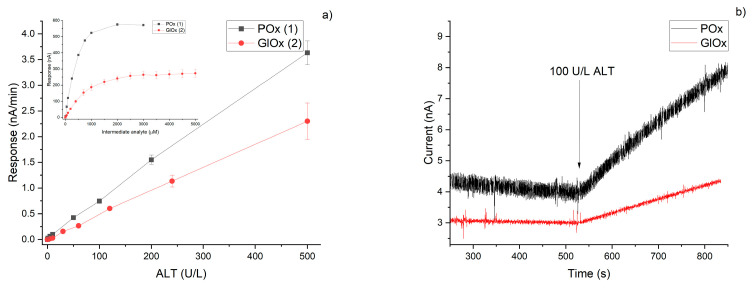
Calibration curves of the developed amperometric biosensors based on POx (1) and GlOx (2) toward intermediate analyte concentrations and ALT activity (**a**). Typical response of the biosensors based on POx (1) and GlOx (2) towards 100 U/L ALT (**b**).

It is well known that the final stage of biosensor development involves the investigation and analysis of its analytical characteristics. These parameters make it possible to assess the applicability of the developed biosensor for detecting the target analyte in relevant biological fluids and within clinically meaningful concentration ranges. Both biosensors developed in this study demonstrated wide working ranges for the determination of the intermediate analyte (pyruvate or glutamate) (Figure 9a, Table 1). The real amperometric responses of the biosensors towards hydrogen peroxide, pyruvate, and glutamate are shown in the Supplementary Figure S5. The ALT detection ranges for both biosensors were also comparable and sufficiently broad (approximately 1–500 and 5–500 U/L ALT for the systems based on POx and GlOx, respectively) (Figure 9b, Table 1), thus enabling the detection of both normal (30 U/L [5]) and elevated (up to 15 times higher [34]) ALT levels in blood serum with either biosensor accounting for 10-times dilution.

**Figure 10:**
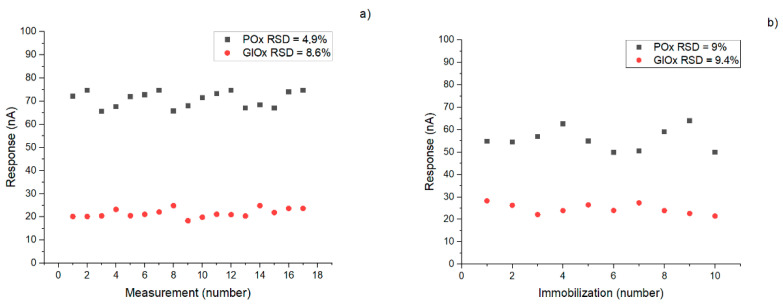
Reproducibility of biosensor responses to 0.1 mM of intermediate analyte (pyruvate or glutamate for POx- and GlOx-based biosensors respectively) under continuous operation (**a**), and reproducibility of the immobilization procedure for POx- and GlOx-based biosensors (**b**).

Reproducibility of responses is critically important in biosensorics, as it ensures signal stability and enables repeated measurements with minimal variability. Similarly, reproducibility of biological material immobilization plays a key role not only for sensor reliability but also for scalability and commercial translation of the technology. Both biosensors exhibited acceptable values for these parameters (within 10%) (Figure 10); however, the POx-based system proved to be slightly more stable compared to the GlOx-based one. Storage stability was preliminarily evaluated for the intermediate analytes, showing that after two months of storage in a dry frozen state, the POx-based biosensor retained 40% of its activity, while the GlOx-based biosensor retained 70%.

## 4. Discussion

A comparative evaluation of the two developed biosensor designs demonstrated that both systems—based on pyruvate oxidase (POx) and glutamate oxidase (GlOx)—are suitable for alanine aminotransferase (ALT) detection; however, their distinct properties may determine the most appropriate applications in clinical diagnostics or commercialization.

The GlOx-based biosensor was characterized by a simpler configuration, as it did not require additional cofactors or auxiliary substrates for the functioning of the bioselective element, in contrast to the POx-based system. Combined with considerably lower optimal substrate concentrations for ALT, this makes the GlOx biosensor less sensitive to changes in sample composition and more cost-effective. Furthermore, the influence of protein matrices on the GlOx-based sensor was smaller, which enhances its applicability for analyzing complex samples, including blood serum and plasma. An additional unique advantage of the GlOx system lies in its versatility: since its response is based on glutamate detection, the platform can be reconfigured for aspartate aminotransferase (AST) measurement by substituting the input substrates. Such flexibility is particularly valuable in the context of commercialization, where there is a growing demand for universal sensing platforms capable of adapting to multiple clinically relevant enzymes.

In contrast, the POx-based biosensor required the presence of several cofactors (Mg^2+^, thiamine pyrophosphate, phosphate ions), which complicates its operation and increases reagent costs. This requirement may explain the differences in optimal ALT substrate concentration ratios observed between the two designs of biosensors: magnesium complexation with amino acid substrates likely reduces their availability for the ALT active site, thereby necessitating higher working concentrations. The stronger negative effect of protein structures in solution on the POx-based biosensor compared to the GlOx-based biosensor may be attributed to a denser polymeric membrane that is more sensitive to diffusion-related changes, as well as to protein interactions with cofactors that lower their availability for the enzyme. Nevertheless, the POx-based sensor demonstrated higher sensitivity, a lower detection limit, and a wider linear range (Table 1), enabling more accurate determination of both physiological and pathological ALT levels in serum. Importantly, it also allowed signal differentiation between ALT and AST, thus opening opportunities for multiplexed systems for the simultaneous detection of multiple aminotransferases and improved diagnostic specificity.

**Figure 11:**
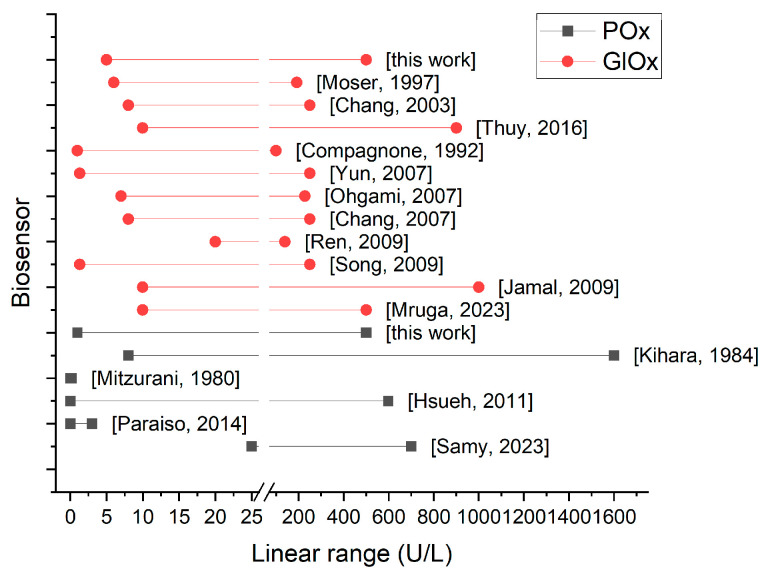
Linear ranges of reported amperometric biosensors for ALT. [14,15,16,35,36,38,39,40,41,42,43,44,45,46,47,48].

When comparing our results with other amperometric biosensors for ALT detection, the following trends were observed:

- Bioselective elements based on POx were mainly constructed using entrapment or adsorption methods [14,35,36,37,38,39], whereas most GlOx-based systems relied on covalent cross-linking [14,15,40,41,42,43,44,45,46], with adsorption or entrapment being less common [16,47,48,49].
- The reported limits of detection for POx-based biosensors (2.97 [35], 2.86 × 10^−4^ [36], 2.18 [14]) were generally slightly lower than those for GlOx-based ones (2.5 [46], 8.48 [16], 3.29 [15], 0.2 [45]).
- The linear ranges of POx-based biosensors were either very narrow at ultralow concentrations (notably in 0.0005–0.18 oxygen [38] and 0.0003–3.0 U/L graphite [36] electrode systems) or wider than those observed for GlOx-based biosensors (Figure 11).
- The response times of POx-based sensors were generally faster (20–240 s) compared with GlOx-based sensors (one reported 5 s, one 10 s, while the majority ranged from 100 to 900 s).

Therefore, the choice between POx- and GlOx-based bioselective elements for biosensoric-ALT determination depends on the type of real samples targeted, the expected concentration ranges, the number of measurements (influenced by sensor stability), and other operational parameters. POx-based systems provide superior analytical characteristics and the possibility to distinguish ALT from AST signals, making them well-suited for integrated diagnostic platforms. GlOx-based systems, although less sensitive, are more economical, less prone to protein interference, and adaptable for AST detection, which makes them attractive for routine diagnostics and commercial implementation. Nevertheless, for both systems it would be advantageous to apply additional signal amplification strategies and approaches to enhance the storage stability of the bioselective material.

## 5. Conclusions

A comparative analysis of POx- and GlOx-based biosensors for ALT detection demonstrated that both designs are effective but differ in their advantages. The POx-based system exhibited higher sensitivity, lower detection limits, and a broader linear range, enabling precise quantification of ALT across physiological and pathological levels and allowing signal differentiation from AST. In contrast, the GlOx-based biosensor offered simpler operation without auxiliary cofactors, lower dependence on substrate concentrations, reduced interference from protein matrices, and the potential to be adapted for AST detection, making it cost-effective and versatile.

Thus, POx-based biosensors are better suited for applications requiring high analytical performance and multiplexed detection, whereas GlOx-based systems are more advantageous for routine diagnostics and commercialization due to their robustness, economy, and adaptability.

## Figures and Tables

**Table 1:** Analytical characteristics of POx- and GlOx-based biosensors.

	Parameter	POx-Based Biosensor	GlOx-Based Biosensor
Intermediate analyte	Sensitivity, nA/mM	573 ± 8	186 ± 19
	Linear range, μM	10–750	10–700
	LOD, μM	0.25 ± 0.1	1.39 ± 0.2
	Dynamic range, μM	1–2000	1–3000
	Immobilization reproducibility error, %	9	9.4
	Response reproducibility error, %	4.9	8.6
	Baseline current, nA	4.1 ± 1.8	3.18 ± 0.05
	Baseline noise, nA	0.20 ± 0.12	0.17 ± 0.04
	Response noise, nA	0.61 ± 0.28	0.19 ± 0.04
	Response time, s	55 ± 11	19 ± 5
	Analysis duration, s	700	700
ALT	Sensitivity, nA/min at 100 U/L	0.75	0.49
	Linear range, μM	1–500	5–500
	LOD, μM	1	1

## Data Availability

The dataset is available on request from the authors.

## Supplementary Materials

The following supporting information can be downloaded at: https://www.mdpi.com/article/10.3390/mi16101168/s1. Figure S1: Bare electrode, 25 keV; Figure S2: Electrode with PPD membrane, 4 keV; Figure S3: Enzyme structure on the electrode surface, 25 keV; Figure S4: Biosensor response to 100 µM to H_2_O_2_ on different potentials (a). Biosensor voltammograms before (black line) and after (red line) 100 µM to H_2_O_2_ addition (b); Figure S5: Typical biosensor responses to the addition of 100 µM of analyte.

## Abbreviations

The following abbreviations are used in this manuscript:

POx	Pyruvate oxidase
GlOx	Glutamate oxidase
ALT	Alanine aminotransferase
AST	Aspartate aminotransferase
PVA-SbQ	Poly(vinyl alcohol)-quarternized stilbazole
BSA	Bovine serum albumin
LOD	Limit of detection
PLP	Pyridoxal phosphate
TPP	Thiamine pyrophosphate
HEPES	N-[2-Hydroxyethyl]piperazine-N′-[2-ethanesulfonic acid]
RT	Room temperature
