# Supplementary material for: Development and Comparative Evaluation of Two Enzyme-Based Amperometric Biosensor Designs for Alanine Aminotransferase Determination in Biological Fluids
Source: Micromachines (Basel). 2025 Oct 15;16(10):1168. doi: 10.3390/mi16101168 (PMC12566202; doi:10.3390/mi16101168)
Supplement: Supplementary file 1 [file micromachines-16-01168-s001.zip › micromachines-3874540-supplementary.pdf]

SEM photo revealed that:

Bare electrode (Fig. S1) has a polished platinum surface and displays a uniform metallic texture without additional structures.

Electrode with PPD membrane (Fig. S2) has a thin organic polymer layer (<2 μm) that uniformly covers the electrode and surrounding glass surface.

Electrode with enzyme membrane (Fig. S3) has needle-like organic structures formed by the enzyme–crosslinker matrix, with a membrane thickness of ~900 nm.

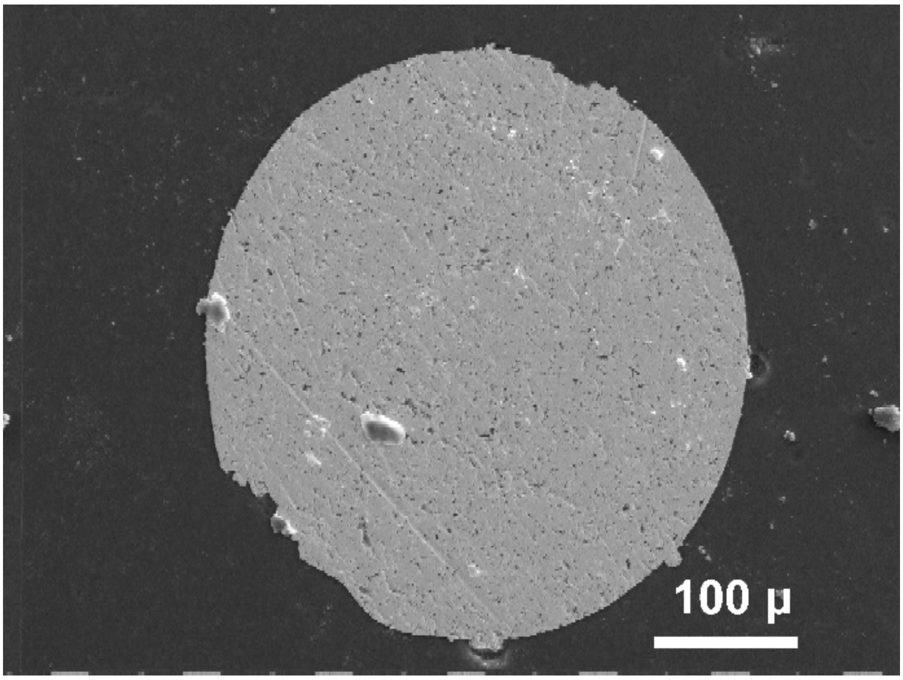

Figure S1. Bare electrode, 25 keV.

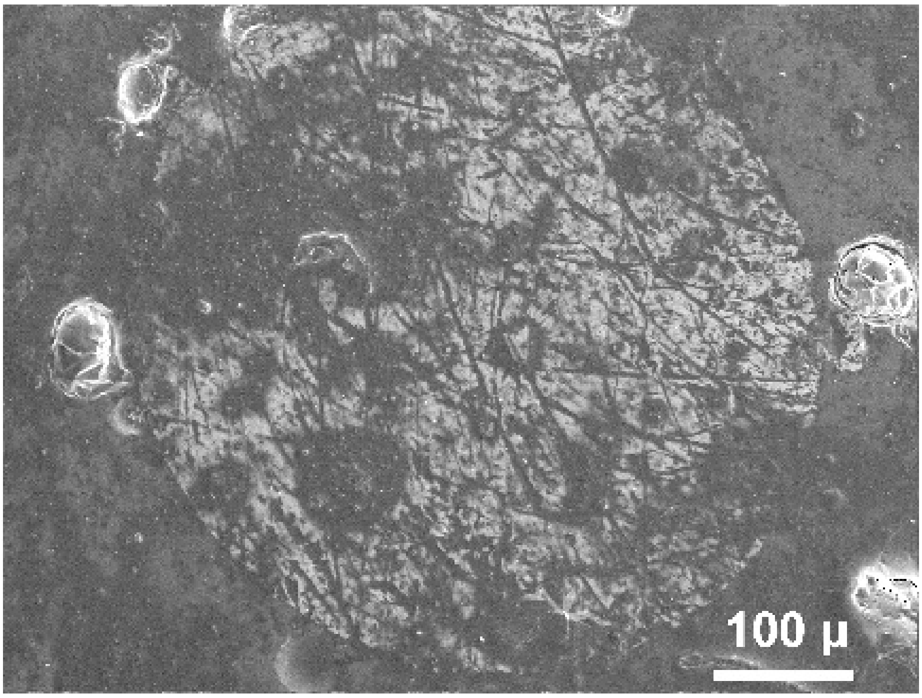

Figure S2. Electrode with PPD membrane, 4 keV.

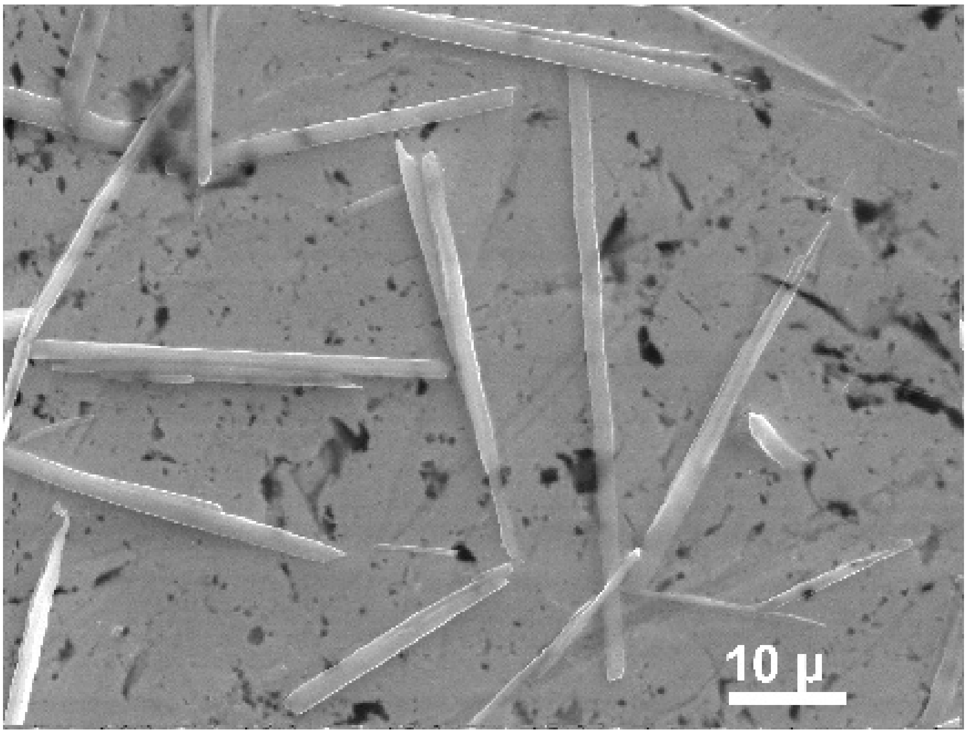

Figure S3. enzyme structure on the electrode surface, 25 keV.

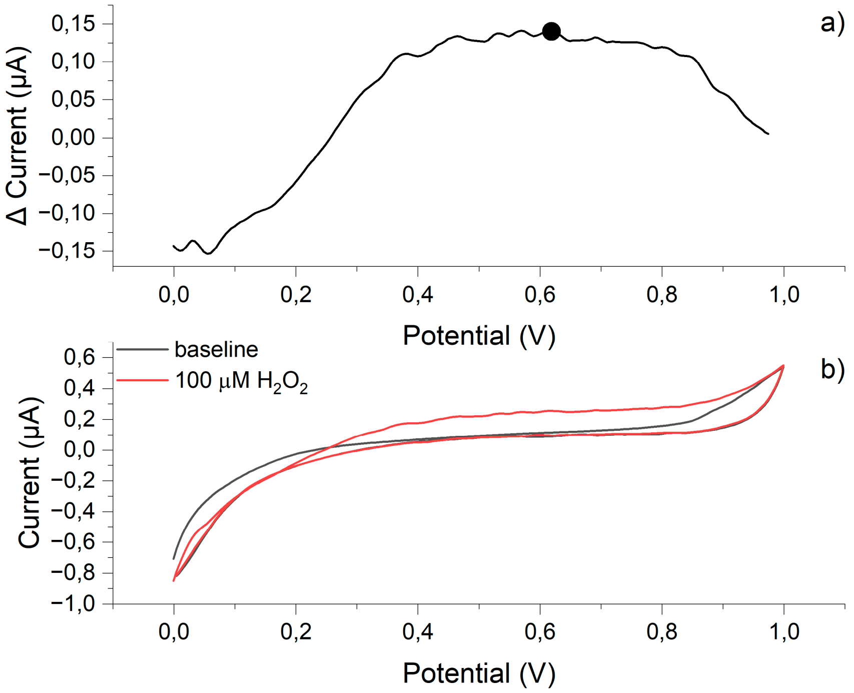

Figure S4. Biosensor response to 100 µM to H_2O_2 on different potentials (a). Biosensor voltammograms before (black line) and after (red line) 100 µM to H_2O_2 addition (b).

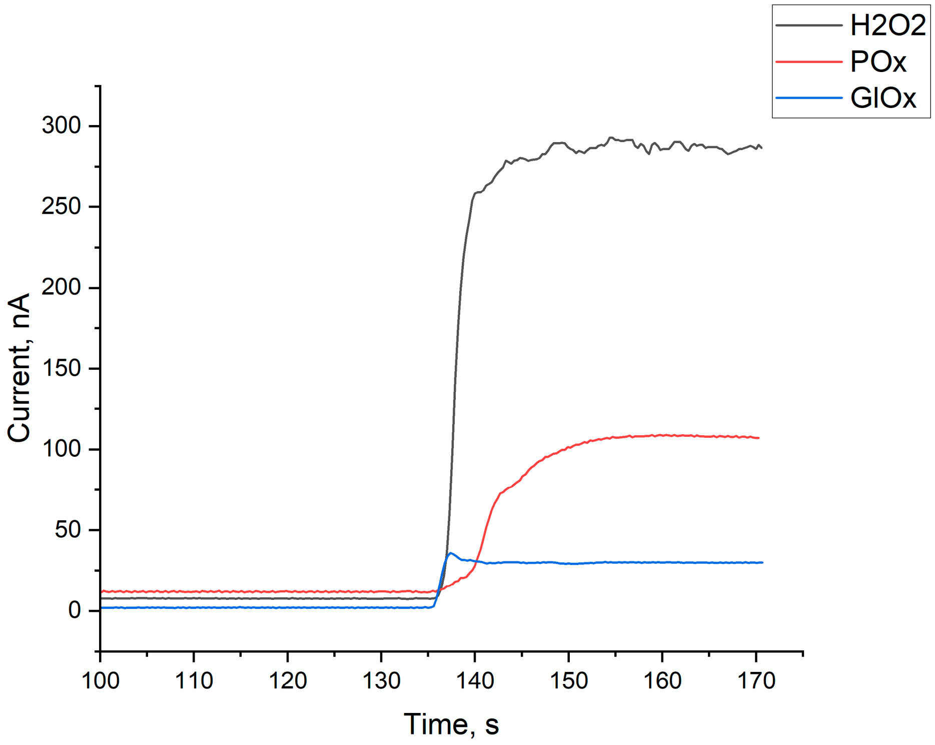

Figure S5. Typical biosensor responses to the addition of 100 μM of analyte.
